# State-space models reveal a continuing elephant poaching problem in most of Africa

**DOI:** 10.1038/s41598-020-66906-w

**Published:** 2020-06-23

**Authors:** Scott Schlossberg, Michael J. Chase, Kathleen S. Gobush, Samuel K. Wasser, Keith Lindsay

**Affiliations:** 1 Elephants Without Borders, PO Box 682, Kasane, Botswana; 2 Center for Conservation Biology, Department of Biology, University of Washington, Seattle, WA 98195 USA; 3 Amboseli Trust for Elephants, PO Box 15135, Langata, Nairobi 00509 Kenya

**Keywords:** Conservation biology, Zoology, Conservation biology

## Abstract

The most comprehensive data on poaching of African elephants comes from the Monitoring the Illegal Killing of Elephants (MIKE) program, which reports numbers of illegally killed carcasses encountered by rangers. Recent studies utilizing MIKE data have reported that poaching of African elephants peaked in 2011 and has been decreasing through 2018. Closer examination of these studies, however, raises questions about the conclusion that poaching is decreasing throughout the continent. To provide more accurate information on trends in elephant poaching, we analyzed MIKE data using state-space models. State-space models account for missing data and the error inherent when sampling carcasses. Using the state-space model, for 2011–2018, we found no significant temporal trends in rates of illegal killing for Southern, Central and Western Africa. Only in Eastern Africa have poaching rates decreased substantially since 2011. For Africa as a whole, poaching did decline for 2011–2018, but the decline was entirely due to Eastern African sites. Our results suggest that poaching for ivory has not diminished across most of Africa since 2011. Continued vigilance and anti-poaching efforts will be necessary to combat poaching and to conserve African elephants.

## Introduction

Beginning around 2007, a wave of poaching for ivory affected populations of savannah elephants (*Loxodonta africana*) and forest elephants (*L. cyclotis*) across Africa^1^. The total population of savannah elephants decreased by 30% between 2007 and 2015^2^, and an estimated 100,000 elephants of both species were poached between 2010 and 2012^3^. In some countries, elephant populations declined by over 50% in under 10 years^2^. With elephant populations and ranges already greatly reduced from pre-colonial levels, such losses put many populations at risk of extirpation^4,5^.

Recent reports, however, indicate that elephant poaching may be abating^6,7^. Since 2016, some African parks have reported reductions or even a halt in elephant poaching^8,9^. Likewise, global ivory prices appear to have peaked and may have begun to fall, perhaps as a result of bans on ivory sales^10^. Accurately determining whether or not poaching is diminishing is critical for evaluating the success of ivory trade bans and other anti-poaching measures. Controversially, several African countries have proposed selling stockpiles of ivory^11^. Such sales may not be justifiable if elephant poaching is continuing at the high levels of the early 2010s.

Elephant population surveys tend to be infrequent, so our main source of information on poaching rates is the Monitoring the Illegal Killing of Elephants (MIKE) program, administered by the Convention on the International Trade in Endangered Species of Wild Fauna and Flora (CITES). Accordingly, rangers at sites across African gather data on the cause of death for elephant carcasses encountered during patrols^12^. The proportion of carcasses killed illegally, as opposed to deaths due to natural causes, legal hunting, or killing of problem animals by wildlife authorities, is known as “PIKE” and is considered an index of poaching rates^3^. PIKE data are typically aggregated to estimate regional or continental poaching rates. For all of Africa, estimates using the MIKE program’s methodology show a 31% reduction in PIKE between 2011 and 2018 (see Results). The program recently reported that PIKE has exhibited a “steady downward trend” since 2011^6^.

CITES estimates PIKE values for Africa as a whole via general linear models, treating region and year as fixed effects so that

1 $$\begin{array}{c}PIK{E}_{i,t}=regio{n}_{i}+yea{r}_{t}+{{\epsilon }}_{i,t}\end{array}$$

where *i* indexes site, *t* is year, and ${{\epsilon }}_{i,t}$, is a normal error term with mean = 0^13^. In the models, observations are weighted by the total number of carcasses (legal and illegal) reported for that site and year. Per the MIKE program, this weighting ensures that sites with better sampling, defined as those with more carcasses reported, have the most influence on PIKE estimates^13^.

These models, while simple, pose several significant problems that call into question the resulting PIKE estimates and inferences about trends in poaching. First, weighting observations by observed numbers of carcasses leads to biased inferences. Ideally, carcass sample sizes would be directly proportional to the number of live elephants in each ecosystem so that the resulting PIKE estimates would be an accurate index of overall poaching rates. In reality, reported carcasses have a weak relationship to elephant population sizes (r^2^ = 0.21; unpublished data), so the resulting PIKE estimates are biased compared to the true poaching rate. Also, because observed numbers of carcasses fluctuate by year, the weights of the sites also vary from year to year. As a result, changes in PIKE estimates from year to year are not entirely due to changes in poaching rates. Another problem with the MIKE analyses is missing data. For 2003–2018, an average of 27% of African MIKE sites failed to report any carcasses. Missing data are not random, either, as many sites are missing data for continuous blocks of years or alternating years, and some sites report results more consistently than others. The model in equation (1) does not account for missing data, so PIKE estimates may be biased by changing composition of the sample. Finally, linear modeling of PIKE values assumes that errors are normally distributed. PIKE, however, is on a [0–1] probability scale, which violates the normality assumption of general linear models and results in biased confidence intervals^14^.

These problems with the CITES MIKE analysis^13^ make it difficult to know if elephant poaching is actually decreasing. As an alternative, we suggest using state-space models to assess trends in PIKE and elephant poaching. With state-space models, the observed PIKE values are considered a noisy sample from the state, which is the “true” underlying value of PIKE^15^. Change in the state from year to year can be modeled as a parametric process such as a random walk. Compared to observed PIKE, state values are smoothed and relatively insensitive to stochastic fluctuations in observed numbers while still tracking real changes in PIKE. In the state-space model, observed PIKE values are a binomial sample from the underlying state. Because binomial sampling has inherent error, observed PIKE values will deviate from the state values. The amount of smoothing is inversely proportional to the number of carcasses observed. State-space models can be fit to MIKE data via the extended Kalman filter^16^. Using state-space models with MIKE data deals with all of the problems mentioned above and should produce a more accurate index of poaching rates. Here, we used state-space models to assess recent trends in poaching in Africa and determine whether or not poaching has declined in recent years, both for the continent and by region.

## Results

**Figure 1:**
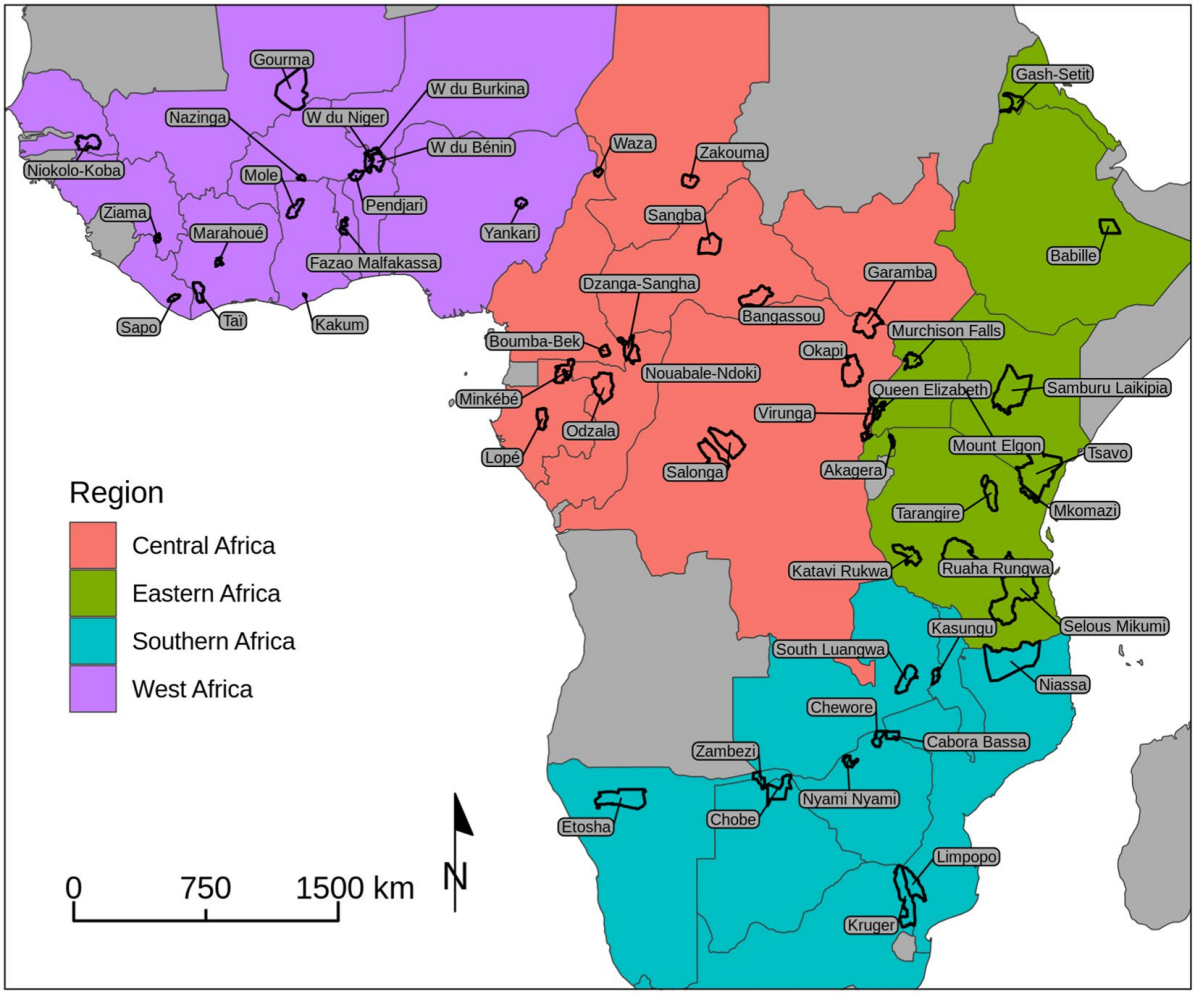
MIKE sites by region in Africa, with site names labelled. Sites are outlined in black. This map was created in Program R^27^ using Natural Earth data (https://www.naturalearthdata.com) and CITES MIKE data (https://www.cites.org/eng/prog/mike/index.php).

Of the 53 African MIKE sites (Fig. 1), 38 sites from 28 countries had carcass data for ≥4 years in 2003–2011 and ≥4 years in 2011–2018 and were included in the state-space models. The 38 sites had a mean of 13.1 years of data (range: 9–16 years) for the 16-year study period and averaged 31.3 carcasses reported per year (range: 3.1–187.7). Estimated live elephant populations averaged 4,845 on the 38 included sites and 319 on the 15 excluded sites. Consequently, excluding these sites should have little effect on our conclusions because excluded sites’ weights would be small when calculating regional estimates. Numbers of carcasses reported and estimated elephant population sizes varied substantially by region and were generally largest in Eastern and Southern Africa and smallest in West Africa (Table 1).

**Table 1:** Sample sizes and summary statistics for MIKE sites used in the state-space analysis. Values other than the number of sites are shown as the mean with range by site in parentheses.

Region	Sites	No. years with data			No. carcasses	Est. no. live elephants
		2003–2011	2011–2018	2003–2018		
Central Africa	12	7.3 (4–9)	6.8 (4–8)	13.1 (9–16)	17.8 (5.7–59.0)	3,529 (213–18,844)
Eastern Africa	10	8.3 (7–9)	7.5 (5–8)	14.9 (12–16)	51.0 (3.8–187.7)	6,559 (105–20,619)
Southern Africa	9	7.9 (4–9)	8.0 (8–8)	14.9 (11–16)	46.1 (12.5–130.1)	8,078 (1,338–27,802)
West Africa	7	5.9 (5–8)	6.6 (6–8)	11.7 (10–15)	7.5 (3.1–17.4)	492 (35–1,003)
Africa	38	7.4 (4–9)	7.2 (4–8)	13.7 (9–16)	31.3 (3.1–187.7)	4,845 (35–27,802)

Using the state-space model, we estimated “sPIKE,” the smoothed, state estimate of PIKE, for each site and year. In general, sPIKE values deviated little from raw PIKE where the number of carcasses was large, but discrepancies were greater when the number of carcasses was small (Supplementary Fig. S1). This was expected because binomial sampling error is inversely proportional to sample size. For a few sites, sPIKE estimates were flat over time, suggesting that observation error explains most or all of the temporal variation in PIKE. Models showed a good fit to the data (r = 0.93) and no significant spatial or temporal autocorrelation in residuals (Supplementary Fig. S2).

**Figure 2:**
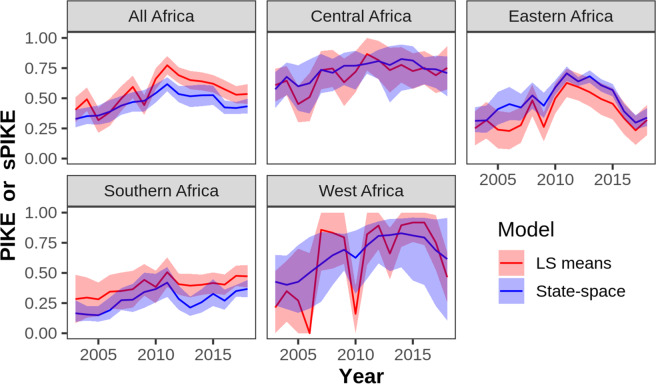
Smoothed sPIKE estimates from state-space models and PIKE estimates from CITES least-squares means (“LS means”) model for all of Africa and by subregion. Lines indicate mean estimates, and shading indicates 95% confidence intervals.

For the continent as a whole, the state-space model predicted consistently lower poaching levels than the least-squares mean estimates from CITES (Fig. 2). By region, however, differences between the CITES and state-space models were more idiosyncratic. In Central and Eastern Africa, sPIKE values were greater than the CITES values (Fig. 2). By contrast, sPIKE estimates in Southern Africa were lower than the CITES estimates. In West Africa, confidence intervals on sPIKE were wide for most years due to small sample sizes ($\bar{x}$ = 7.5 carcasses site^−1^ year^−1^).

Changing the minimum sample size requirements for 2003–2011 and 2011–2018 had little effect on our results. Other than small differences in confidence intervals, sPIKE estimates were nearly identical for minimum samples of 2–5 years in each time period (Supplementary Fig. S3). When we increased the minimum sample size to 6, we obtained moderately lower sPIKE estimates. Only 26 sites met the criteria for inclusion with a minimum of 6 observations in each time period.

For 2003–2010, sPIKE for the entire continent increased at a significant rate of 0.03 year^−1^ (Table 2). Trends in sPIKE were increasing and significant in all four regions for those years. For 2011–2018, sPIKE decreased significantly in eastern Africa and for Africa as a whole (Table 2). Trend estimates for Southern and Western Africa were small and not significantly different from 0. The trend for Central Africa was negative but not significant after Bonferroni correction.

**Table 2:** Estimated trends in sPIKE from state-space models by region and time period. Trends in bold were significant after Bonferroni correction.

Region	Years	Estimate ± SE	t	P
Entire continent	**2003–2010**	**0.03 ± 0.002**	**13.40**	**<0.001**
	**2011–2018**	**−0.03 ± 0.004**	**−6.13**	**<0.001**
Central Africa	**2003–2010**	**0.03 ± 0.01**	**4.42**	**0.004**
	2011–2018	−0.01 ± 0.004	−2.54	0.04
Eastern Africa	**2003–2010**	**0.04 ± 0.01**	**6.11**	**0.001**
	**2011–2018**	**−0.06 ± 0.01**	**−6.35**	**0.001**
Southern Africa	**2003–2010**	**0.03 ± 0.006**	**5.80**	**0.001**
	2011–2018	0.01 ± 0.01	0.64	0.55
West Africa	**2003–2010**	**0.05 ± 0.01**	**6.89**	**<0.001**
	2011–2018	−0.003 ± 0.01	−0.27	0.80

We used simulations to test the accuracy of the state-space model. With completely random PIKE values, continental sPIKE estimates had mean root mean square error (rmse) of 0.04 (range: 0.02–0.08). By site, rmse averaged 0.20 (range: 0.18–0.23). For simulations with logit-linear trends in PIKE, the mean continent-wide rmse was lower at 0.02 (range: 0.01–0.03). By site, rmse for simulations with trends averaged 0.10 (range; 0.08–0.13).

## Discussion

Analyzing MIKE data with state-space models, we found no significant trends in sPIKE for 2011–2018 in three of four African regions. Only in Eastern Africa did we find a significant trend, a clear decrease in sPIKE for 2011–2018. Southern, Central, and Western Africa all had non-significant trends in sPIKE for those years. Even if we accept the result for Central Africa as significant, the trend was just −0.01 year^−1^, a rate of change just 15% as large as the trend for East Africa (Table 2). The 2011–2018 trend for the continent as a whole was negative, but the effect is driven entirely by the strong decline in Eastern Africa. To demonstrate this, we recalculated continental sPIKE while excluding data from Eastern Africa. The trend estimate for 2011–2018 was −0.007 ± 0.006 year^−1^, which was not significantly different from 0 (P = 0.30). Thus, we conclude that poaching, as measured by sPIKE, has not been decreasing in most of Africa since 2011. This conclusion stands in contrast to recent analyses of MIKE data^6,7^. Though CITES has consistently noted that there is uncertainty in trend estimates^6^, media reports based on CITES reports and a recent paper by Hauenstein *et al*.^7^ have largely ignored this uncertainty and reported declines in poaching levels since 2011^17,18^. By contrast, our findings show the importance of using appropriate analytic methods to measure trends in poaching rates.

The general linear model currently used by CITES to estimate PIKE has two major problems. First, the model is misspecified. Analyzing proportions with linear models is inappropriate because the residuals violate the assumption of normal errors^14^. The MIKE program’s ad hoc solution to this problem is to truncate confidence intervals at [0,1], which results in biased intervals. As further evidence of the model specification problem, the residuals from the CITES model for the continent were non-normal and showed significant temporal autocorrelation (Supplementary Fig. S4). Second, by using PIKE as the dependent variable, the model treats all observations as equally precise. In reality, the variance of a proportion is inversely proportional to its sample size, and sample sizes varied greatly across MIKE sites. The CITES models do weight observations by total carcasses reported. This weighting, however, does not fully account for variance in the precision of PIKE observations. Imagine that all MIKE carcass counts, legally and illegally killed, were multiplied by ten. The resulting confidence intervals on PIKE estimates should be smaller because of larger sample sizes and reduced sampling error. The linear model that uses proportions as the dependent variable would, however, produce identical results with ten times the sample size because all observations in linear models are assumed to have identical precision.

A recent study by Hauenstein *et al*.^7^ used MIKE data to model correlates of poaching and assess recent trends. This study appropriately utilized the binomial distribution to model PIKE with a generalized linear mixed model. In their paper, trends in predicted poaching rates were driven by the positive effect of ivory prices on poaching as well as random year effects. The authors claim that their model showed strongly decreasing poaching rates since 2011 in each of Africa’s four regions, stating “separating poaching rates by region revealed similar temporal patterns among all regions” (p. 4). Because, however, this paper did not calculate separate year effects for each region, its results may overlook regional differences in poaching rates. To test if trends are similar in the four regions, one would need to run a model that fits year effects separately for each region. Simply looking at either the CITES estimates or the sPIKE estimates in Fig. 2 strongly suggests that the trends in the four regions are dissimilar.

In contrast to the other recent analyses, state-space models proved ideal for analyzing MIKE data on elephant carcasses, for several reasons. First, the model takes into account the inherent error in sampling the causes of elephant mortality. As a result, in West Africa, where relatively few carcasses were reported, confidence intervals around sPIKE estimates were wide, suggesting that we lack sufficient data to precisely estimate poaching levels in that region. More generally, incorporating error in the data generation process is critical when modeling any ecological phenomenon^19^. Another major advantage of the state-space model is the ability to account for missing data. In West Africa, the sudden dips in the CITES PIKE estimates in 2006 and 2010 are largely due to many sites’ missing data for those years (Fig. 2). The state-space model does not produce similar dips because it accounts for missing data. Finally, the state-space model is relatively easy to fit using the extended Kalman filter. R packages such as KFAS, dlm, and walker can be used to fit state-space models.

Simulations with known PIKE values showed that our state-space models can accurately estimate poaching rates. The process error for the state-space model with actual MIKE data averaged 1.2 across the 38 sites. In the simulations, mean process error was 0.8 with trends and 3.1 without trends. Thus, the simulations with trends are likely the best guide for assessing the accuracy of the state-space model. Mean rmse for continental estimates was just 0.02 for simulations with an underlying trend in PIKE. This suggests that the state-space model can produce accurate results even with the missing data and relatively small sample sizes that characterize the MIKE dataset. In the simulations, mean rmse was larger when calculated by site, at 0.10. This is unsurprising, as the total sample sizes for many sites were small, and smoothing produces discrepancies between predicted and observed values. Thus, our results suggest that state-space models will be most useful for calculating regional or continental poaching rates rather than site-level estimates.

One notable result of our study is that continental sPIKE estimates were consistently lower than CITES’ PIKE estimates. A likely reason for this difference is that in CITES’ models, observations are weighted by sample size. Consequently, when poaching increases, the number of carcasses increases as well because elephant mortality from poaching is largely additive, not compensatory^3,7^. This gives sites with more poaching excess weight in the linear models and biases the resulting PIKE estimates high.

Our results come with an important caveat: the state-space model cannot account for inherent bias in the reporting of carcasses. Some MIKE sites consistently reported PIKE values equal to or near 1.0 (Supplementary Fig. S1). These sites could be biasing their reporting towards illegally killed carcasses, perhaps based on investigating intelligence reports^12^. Though state-space models can account for sampling error, they cannot correct biased data on their own. Thus, the state-space model is not a panacea for all sampling issues.

Our findings have major implications for conservation of African forest and savannah elephants. Notably, we find that illegal killing has improved little or even worsened since 2011 in Southern, Western, and Central Africa. The reduction in poaching in East Africa appears to be real and is laudable, but conservationists and governments should not allow improvement in one region to influence their view of what is happening in the rest of Africa. Poaching levels remain high and are likely unsustainable in Central and Western Africa. In Western Africa, most savannah elephant populations are small and isolated, meaning that these populations could be at risk of extirpation^4^. In Central Africa, studies have shown major declines in some elephant populations^5,20^. Recent survey data from Southern Africa is limited, but two major elephant populations in this region are showing worrisome trends. Northern Botswana’s large elephant population has been experiencing a spike in poaching since 2017^21^, and Kruger NP in South Africa has experienced heightened poaching recently as well^6^. Taken together, these findings call for continued vigilance and anti-poaching and anti-trafficking efforts.

The MIKE program is an extremely valuable source of information on the status of elephant populations across Africa and Asia. In many countries, elephant surveys are infrequent, and some governments refuse to release elephant survey data. Thus, MIKE is notable for being the only publicly available source of data for many elephant populations. Proper analysis of MIKE data will help to ensure that managers and decision-makers have accurate information needed to conserve elephants. The MIKE program has recently initiated a process to update their analytic methods^6^. We suggest that state-space models or other methods that account for observation error be used in future analyses of MIKE data.

## Methods

### Study areas

We used data from 53 African MIKE sites that began reporting prior to 2010 (Fig. 1). Additional sites in Asia as well as African sites added to the program in 2018 were not considered here. MIKE sites tend to be protected areas, though some sites are unprotected or include both protected and unprotected areas. The 53 sites average 9,863 km^2^ in area (range: 175–51,027 km^2^) and are divided into four regions (Fig. 1). Habitats on MIKE sites are varied and include savannahs, grasslands, tropical forests, and a variety of other vegetation types. In our analyses, we did not attempt to distinguish between sites with forest or savannah elephants. Forest elephants predominate in MIKE’s Central Africa region, though a few sites in this region hold savannah elephants.

### MIKE data

MIKE data were made available by CITES at http://cites.org/eng/prog/mike/data_and_reports. Each MIKE site reports annual totals of the number of carcasses of all origins encountered and the number of illegally killed carcasses encountered. The program utilizes strict criteria for determining a carcass’ cause of death^22^. In our analyses, we used data from 2003–2018; we excluded data from a few pilot sites in 2002. As mentioned above, no data exists for many site-year combinations, and some MIKE sites have few years with data. To accurately estimate trends in PIKE, we arbitrarily restricted the dataset to sites with at least 4 years with data for 2003–2011 and 4 years with data for 2011–2018. We used 2011 as a dividing point because of reports that 2011 was an inflection point for elephant poaching rates, with distinct trends before and afterwards^6,7^. Thus, good estimates of trends in PIKE require multiple observations before and after 2011. We tested how these sample-size restrictions affected our results, as discussed below.

### Elephant population estimates

To estimate PIKE by region or for the continent, we had to weight PIKE estimates from individual sites. The MIKE program weights individual sites by the number of carcasses reported^13^. As noted above, carcasses reported are only weakly related to elephant population size and more likely reflect search effort as well as rates of poaching and natural mortality. Instead, we weighted site PIKE estimates by live-elephant population size. This should allow for better inferences about regional and continental poaching levels because the resulting PIKE estimates should be representative of the entire region or the continent. We obtained elephant population estimates for MIKE sites from four sources (see Supplementary Table S1): published survey reports, unpublished survey reports, the African Elephant database^23^, and African elephant status updates from IUCN^24,25^. For each survey, we examined study area maps to ensure that survey boundaries were congruent with MIKE site boundaries. Where necessary, we excluded survey strata outside MIKE boundaries from population estimates.

MIKE data are reported annually, but elephant surveys were generally less frequent. Weighting by elephant population size requires estimates for each site and year. Thus, for each site, we used linear interpolation to estimate population sizes between surveys. For years prior to the earliest available survey, we used the earliest survey estimate. For years after the latest elephant survey, we used the latest survey estimate. If only a single elephant population estimate was available, we used that estimate for all years. In our sample, there was a positive correlation between the number of elephant population estimates and the mean population size (r = 0.45). This means that fewer interpolated estimates were generally needed for the sites with the largest weights in the analysis.

### State-space models

We used state-space models to estimate the unobserved, “true” PIKE for each site and year while accounting for missing data and smoothing over fluctuations due to sampling error. The state-space model has two components: an observation model, which treats observed PIKE as a noisy sample of the state, and a process model, which treats change in the state over time as a parametric process. To avoid confusion with observed PIKE values, we refer to the state estimates as “sPIKE”; like PIKE, sPIKE is also on a probability scale. The observation model was a draw from a binomial distribution, with probability equal to sPIKE so that

$${K}_{i,t} \sim {\rm{binomial}}({C}_{i,t},{s}_{i,t})$$

where *s* is sPIKE, *K* is the number of illegally killed carcasses, and *C* is the total number of carcasses reported for site *i* and year *t*. We modeled change in sPIKE over time as a random walk on a logit scale as

2 $$\begin{array}{c}{\rm{l}}{\rm{o}}{\rm{g}}{\rm{i}}{\rm{t}}({s}_{i,t})={\rm{l}}{\rm{o}}{\rm{g}}{\rm{i}}{\rm{t}}({s}_{i,t\text{-}1})+{\epsilon }_{i,t}\end{array}$$

3 $$\begin{array}{c}{\epsilon }_{i,t}\sim N(0,{\sigma }_{i}^{2})\end{array}.$$

Here, ${{\epsilon }}_{i,t}$ is the “disturbance,” the change in the state from year to year. Larger values of ${\sigma }_{i}^{2}$, the process error, allow for more rapid change in the state and more “wiggle” in sPIKE estimates. We estimated process error separately for each site, as discussed below.

The state-space model partitions variance in PIKE between the state process and the observation process. As a result, observed values of PIKE, *K*/*C*, will deviate from sPIKE due to binomial sampling. At the same time, sPIKE should be a more accurate index of poaching levels because it is relatively insensitive to outliers and random fluctuations in observed values. Another advantage of the state-space model is the ability to predict sPIKE in years when data was missing for a site, by estimating disturbances from equations (2) and (3). Regional and continental estimates of sPIKE use estimates from all sites in all years so that resulting values are not biased by missing data.

We used the extended Kalman filter to fit the binomial state-space models. The Kalman filter is an algorithm for estimating the underlying state from noisy observations^16^. In practice, the Kalman filter optimally partitions the variance in observations between the state and observation processes. The extended Kalman filter uses Taylor-series expansion to approximate the binomial distribution as a linear equation, allowing the model to be fit by maximum likelihood. We made inferences from smoothed estimates of the state^16^.

We ran our models using the KFAS package^26^ in Program R^27^. The Kalman filter can be used with multivariate time series, which combine multiple sites. In such models, the process error term is a covariance matrix, so that correlations between sites can be explicitly modeled. We initially tested multivariate models fit by region, with the process error modeled as an unstructured covariance matrix. This formulation allowed for correlations between sites in the random walk disturbances, as might be occur if sites follow parallel trends over time. We compared the multivariate models with models in which process errors were independent for each site. Per Akaike’s Information Criterion, models with independent disturbances by site were strongly preferred over models with correlated disturbances. Thus, we made inferences from models in which disturbances were uncorrelated between sites.

To validate models, we assessed model residuals for spatial and temporal autocorrelation using the ncf package^28^ in R. As a measure of model fit, we computed the correlation between model predictions and actual PIKE values. To test how our minimum sample size requirements affected our results, we used the state-space model to predict continental sPIKE for minima of 2–5 years of data for 2003–2011 and for 2011–2018.

### Regional estimates and trends

We used the site-wise sPIKE estimates to assess trends in poaching for the four regions and for the entire continent. For each year, we calculated regional or continental sPIKE as the weighted mean of the site estimates, with weight equal to estimated elephant populations. Weighting by the number of live elephants is advantageous because the resulting sPIKE estimates should be an index of the overall proportion of elephants poached in the given region. Accordingly,

$$\begin{array}{ccc}{r}_{t} & = & \mathop{\sum }\limits_{i=1}^{N}{w}_{i,t}{s}_{i,t}\\ {w}_{i,t} & = & \frac{{E}_{i,t}}{{\sum }_{i=1}^{N}{E}_{i,t}}.\end{array}$$

Here, *r* is the regional or continental sPIKE estimate, *w* is the weight, *E*_*i,t*_ is the estimated number of live elephants, and *N* is the total number of sites in the given region or continent. We computed variances of regional sPIKE estimates on a logit scale as

$${\rm{V}}({\rm{logit}}({r}_{t}))=\mathop{\sum }\limits_{i=1}^{N}{w}_{i,t}^{2}{\rm{V}}({\rm{logit}}({s}_{i,t}))$$

for two reasons: first, to allow for the normal approximation to hold in calculating confidence intervals, and second, because our state-space models estimate variances for sPIKE on the logit scale. Per Oranje^29^, we calculated 95% confidence limits on regional sPIKE on the logit scale and then back transformed the estimates to the probability scale as

$${{\rm{logit}}}^{-1}[{\rm{logit}}({r}_{t})\pm {z}_{1-\alpha /2}\sqrt{{\rm{V}}({\rm{logit}}({r}_{t}))}].$$

To assess trends, we used linear regression, with regional or continental sPIKE estimates as the dependent variable and year as the independent variable. Because earlier studies showed that poaching peaked in 2011, we conducted separate regressions for 2003–2010 and for 2011–2018 for each region. To account for error in the dependent variable in the regression, we used the feasible generalized least squares method^30^. When the estimated error due to the variance in sPIKE was small, this method was equivalent to inverse-variance weighted least squares regression. To reduce the probability of type-I error, we used Bonferroni correction on the significance levels of the regression coefficients for a family-wise error rate of 0.05.

### Simulations

To test the accuracy of the state-space models, we used simulations with known “true” PIKE values. We ran two sets of simulations, each of which included 100 simulated carcass datasets with sample size identical to that of the MIKE dataset—16 years of observations and 38 sites. In the first set of simulations, we assumed a monotonic trend in actual PIKE values (hereafter “aPIKE”) at each site. For each site, we drew a random starting value for year 1. Subsequent aPIKE values followed a linear trend on a logit scale, with random deviations from the trendline for each year. Accordingly, for site *i*,

$${\rm{logit}}({a}_{i,1}) \sim {\rm{U}}(\,-\,4,\,4)$$

$${\beta }_{i} \sim {\rm{U}}(\,-\,1,\,1)$$

$${\varepsilon }_{i,t} \sim {\rm{N}}(0,1)$$

$${\rm{l}}{\rm{o}}{\rm{g}}{\rm{i}}{\rm{t}}({a}_{i,t|t > 1})={\rm{l}}{\rm{o}}{\rm{g}}{\rm{i}}{\rm{t}}({a}_{i,1})+{\beta }_{i}(t-1)+{\varepsilon }_{i,t}.$$

Here, *a* is aPIKE, *β* is the linear trend in aPIKE on a logit scale, and $\varepsilon$ is the random departure from the trendline. In the simulations, numbers of illegally killed carcasses were randomly drawn for each site and year from binomial distributions with probability = aPIKE and sample size equal to the observed total number of carcasses in the MIKE dataset for that site and year. To make the simulated dataset match the MIKE data, we removed site-year combinations from the simulated dataset that were missing for the actual data. This allowed us to learn how well the state-space model compensated for missing data.

The second set of simulations utilized random values of aPIKE for all sites and years, with no underlying trends. Accordingly,

$${a}_{i,t} \sim {\rm{U}}(0,1)$$

We drew numbers of illegally killed carcasses with binomial draws from the aPIKE values as above. Again, the simulated datasets included only site and year combinations that were not missing in the MIKE dataset.

For each simulated dataset, we used state-space models to estimate sPIKE for each site and year, as described above for the MIKE data. To measure the accuracy of the models, for each set of simulations, we computed the root mean squared error (rmse) of sPIKE for each site and year and averaged the rmse over all estimates. We also calculated continental sPIKE estimates for each simulation and computed the mean rmse over the years for those estimates.

## Supplementary information

Supplementary Information.

## Data Availability

MIKE data used in this study are available at http://cites.org/eng/prog/mike/data_and_reports. Data on elephant population sizes and code used to run the state-space models are included in this published article (and its Supplementary Information files).
