# Supplementary material for: State-space models reveal a continuing elephant poaching problem in most of Africa
Source: Sci Rep. 2020 Jun 23;10:10166. doi: 10.1038/s41598-020-66906-w (PMC7311459; doi:10.1038/s41598-020-66906-w)
Supplement: Supplementary file 1 — Supplementary Information. [file 41598_2020_66906_MOESM1_ESM.pdf]

Supplementary Information

State-space models reveal a continuing elephant poaching problem in most of Africa

Scott Schlossberg[1]*, Michael J. Chase[1], Kathleen S. Gobush[2], Samuel K. Wasser,[2] Keith Lindsay[3]

[1]Elephants Without Borders, PO Box 682, Kasane, Botswana

[2]Department of Biology, University of Washington, Seattle WA 98195, USA

[3]Amboseli Trust for Elephants, PO Box 15135, Langata, Nairobi 00509, Kenya

*email: xanthocephalus@gmail.com

This supplement includes:

Supplemental Figures S1-S4

Supplemental Table S1

R code to run the state-space models

Supplementary Figure S1. sPIKE estimates for (a) Southern Africa, (b) Eastern Africa, (c) Central Africa, and (d) West Africa. Lines are mean sPIKE estimates. Shading indicates 95% confidence intervals on sPIKE. Circles indicate observed PIKE, and circle area is proportional to the observed total number of carcasses.

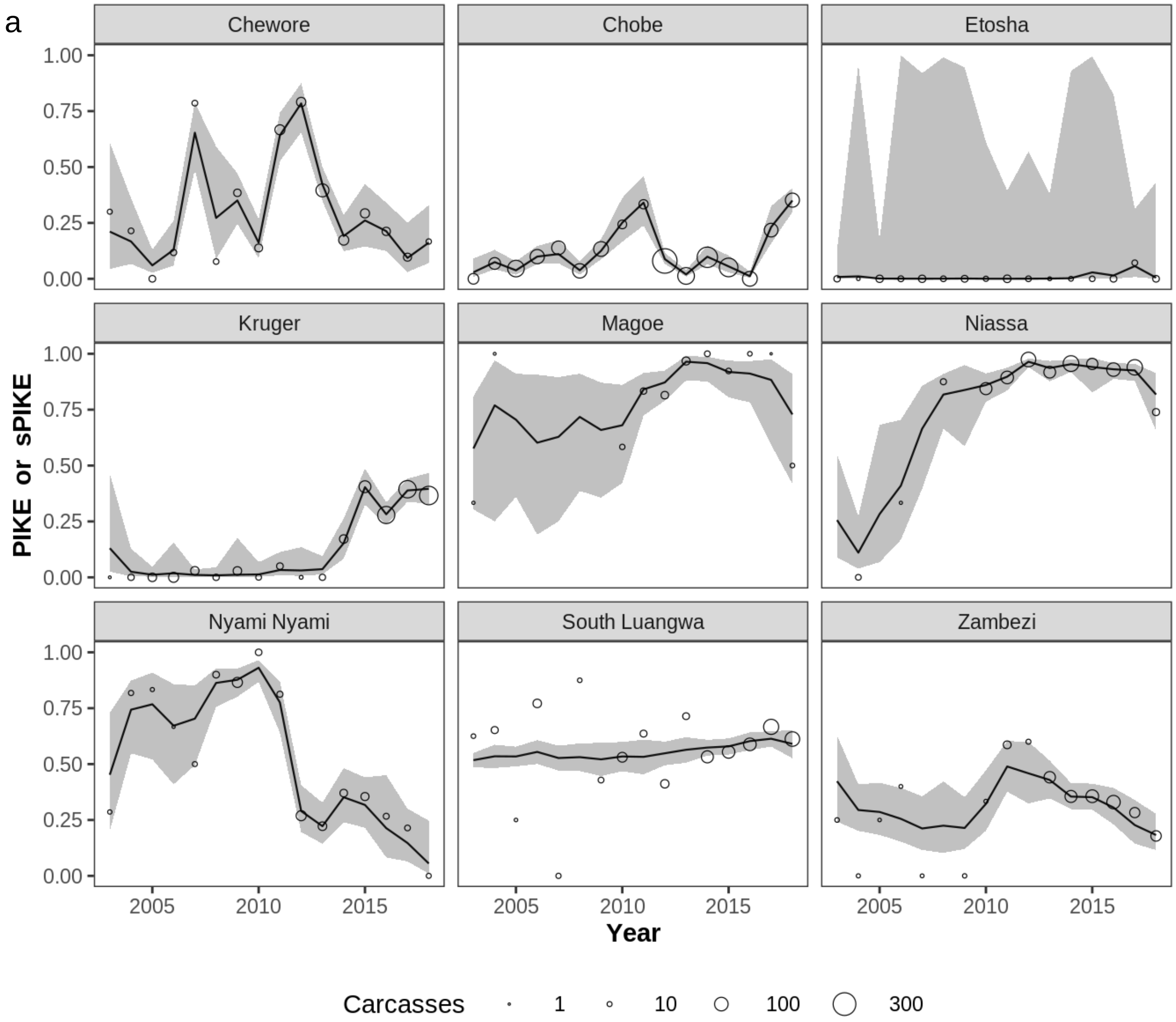

Supplementary Fig. S1 (cont'd)

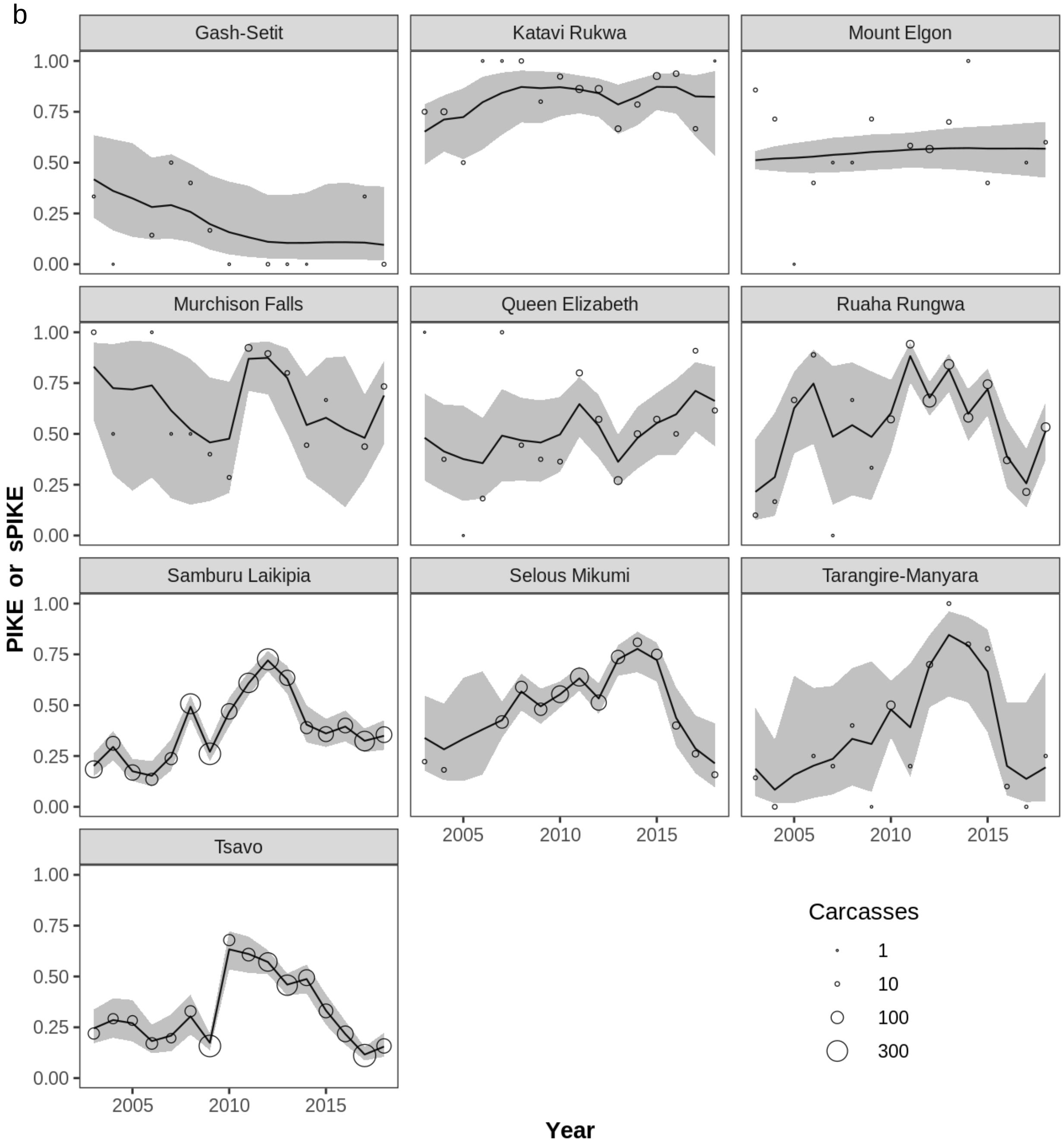

Supplementary Fig. S1 (cont'd)

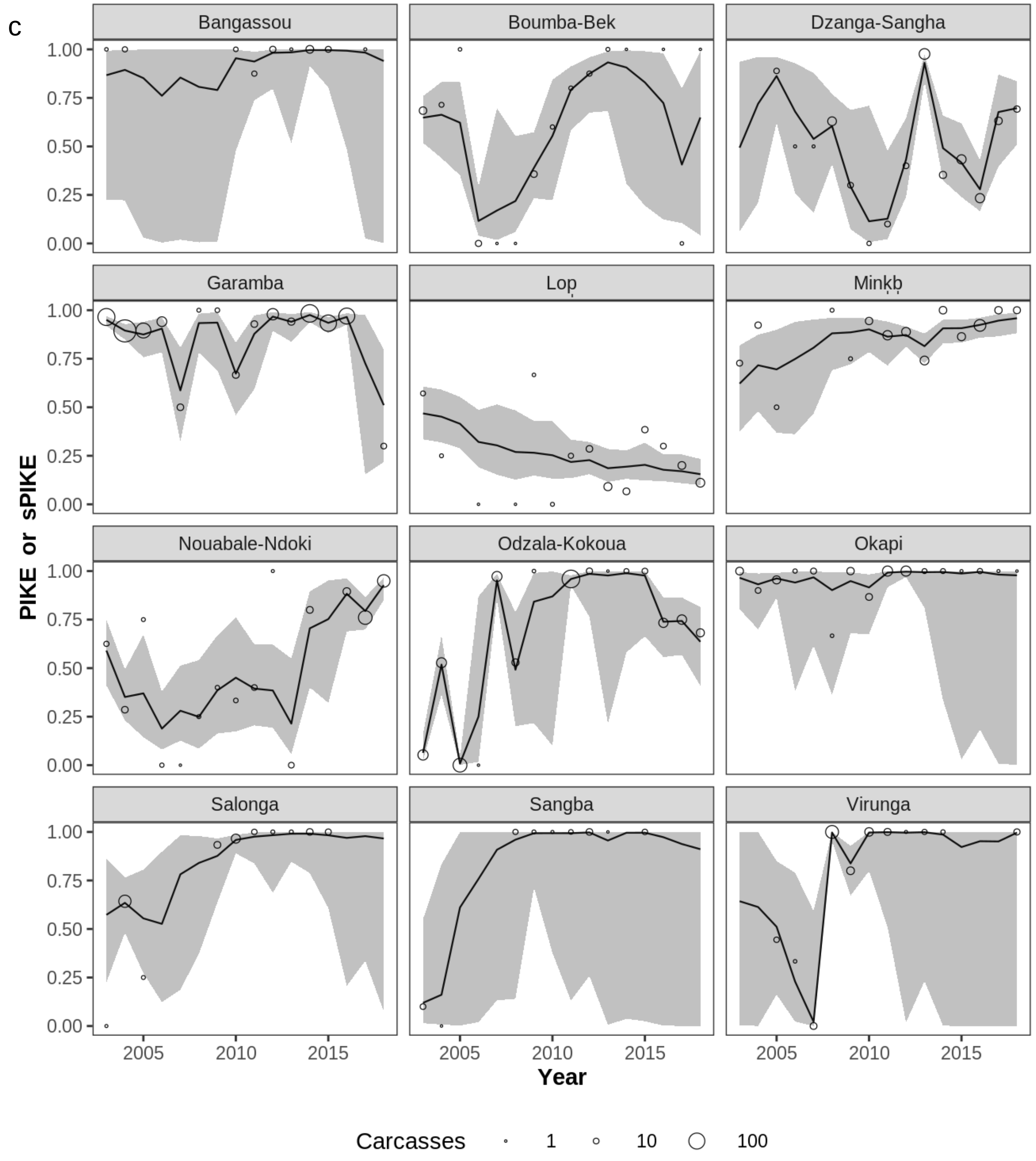

Supplementary Fig. S1 (cont'd)

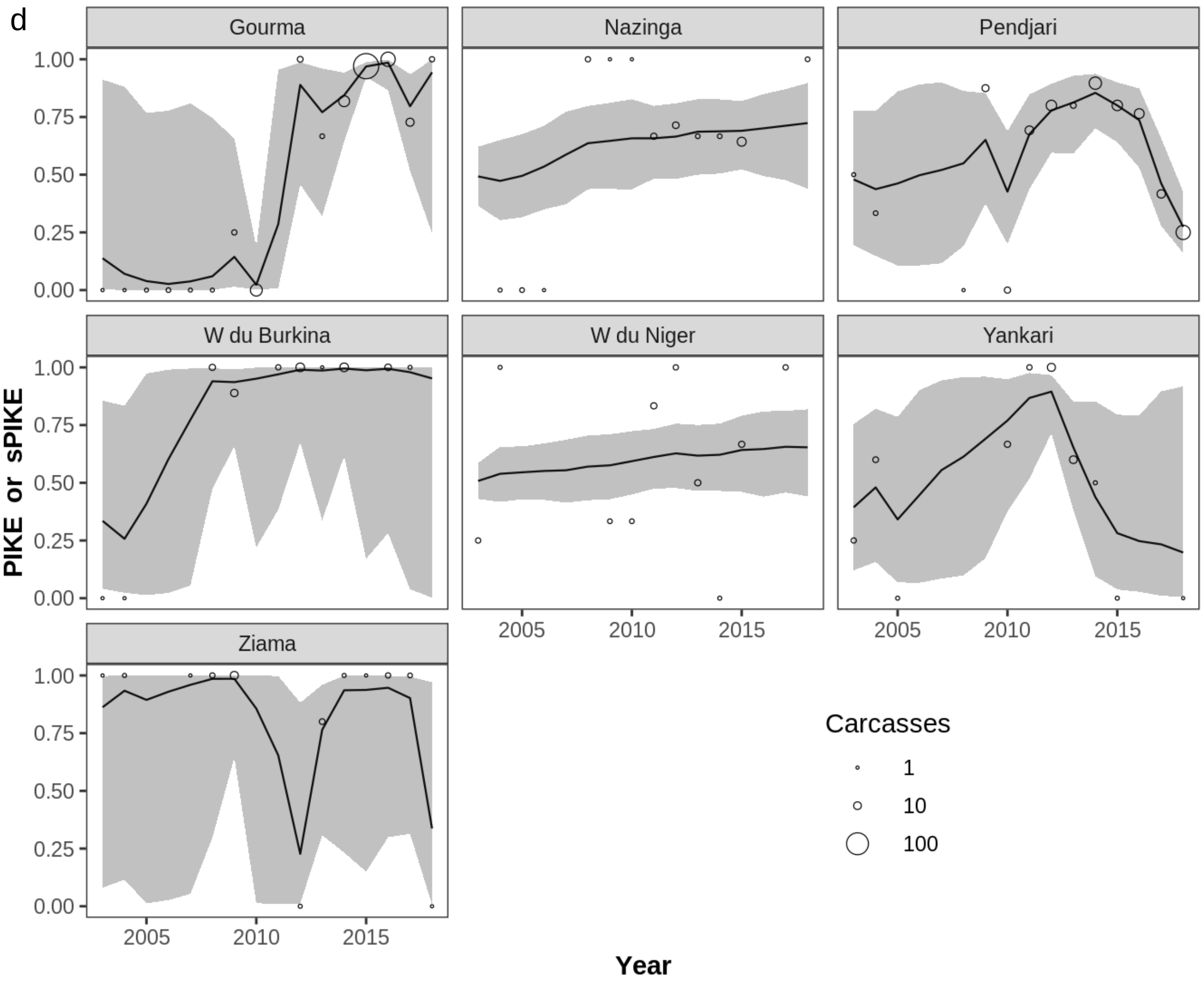

Supplementary Figure S2. (a) Spatial autocorrelation and (b) temporal autocorrelation in residuals of state-space models. Black lines indicate mean autocorrelation, and shading indicates bootstrapped 95% confidence intervals.

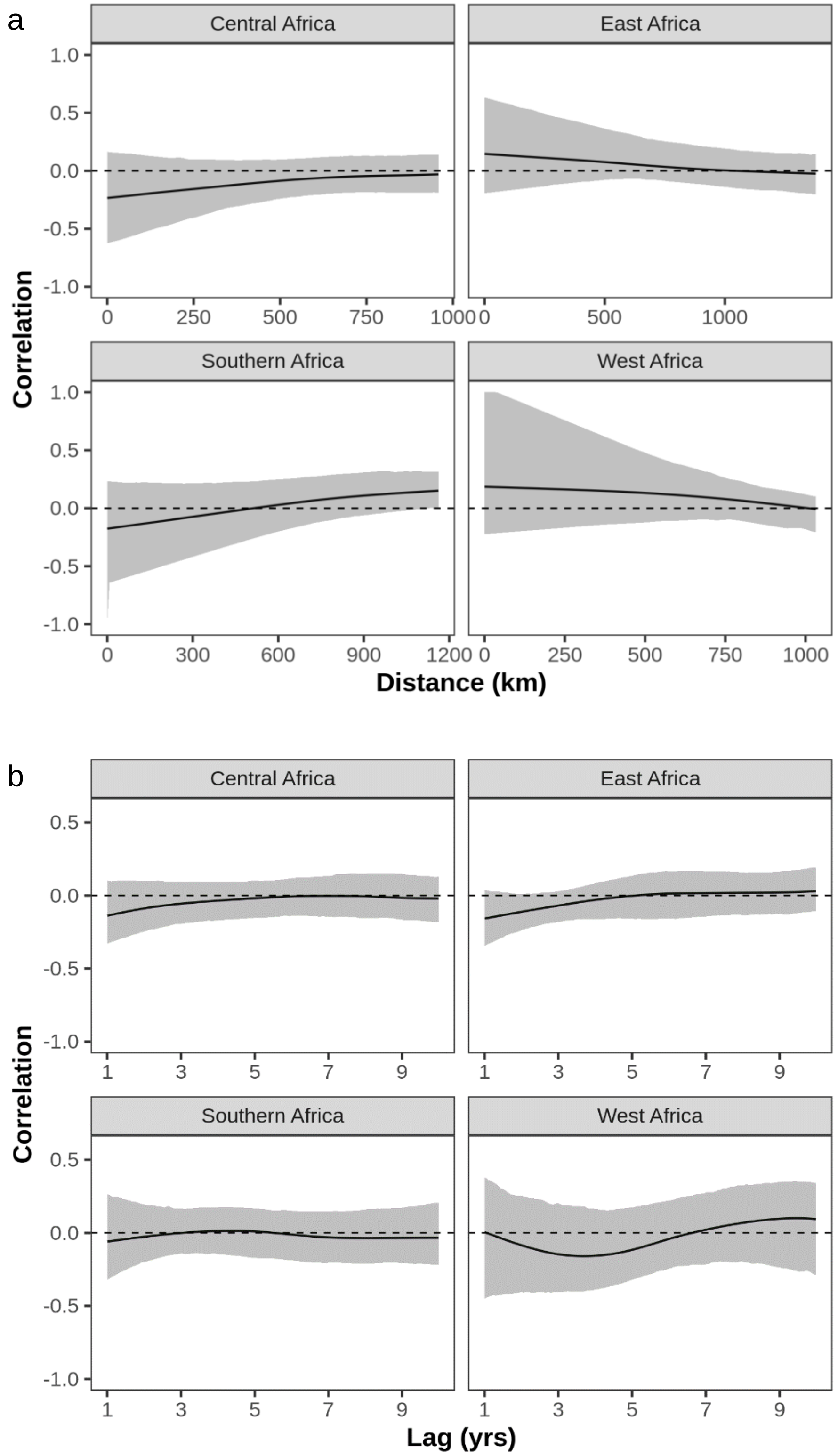

Supplementary Figure S3. Effects of alternate minimum sample sizes on continental sPIKE estimates from the state-space model. Bars indicate mean estimates; error bars indicate 95% confidence intervals.

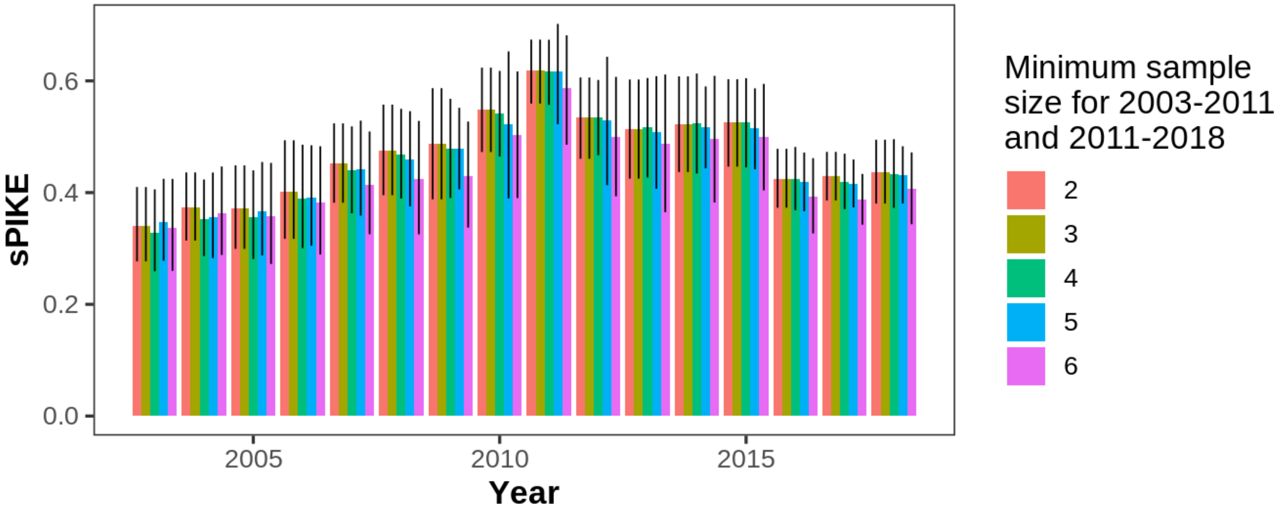

Supplementary Figure S4. (a) Significant temporal autocorrelation and (b) non-normal residuals from the continental least-squares model for PIKE used by CITES. For (b), Shapiro-Wilks test for normality: W = 0.98, P < 0.001.

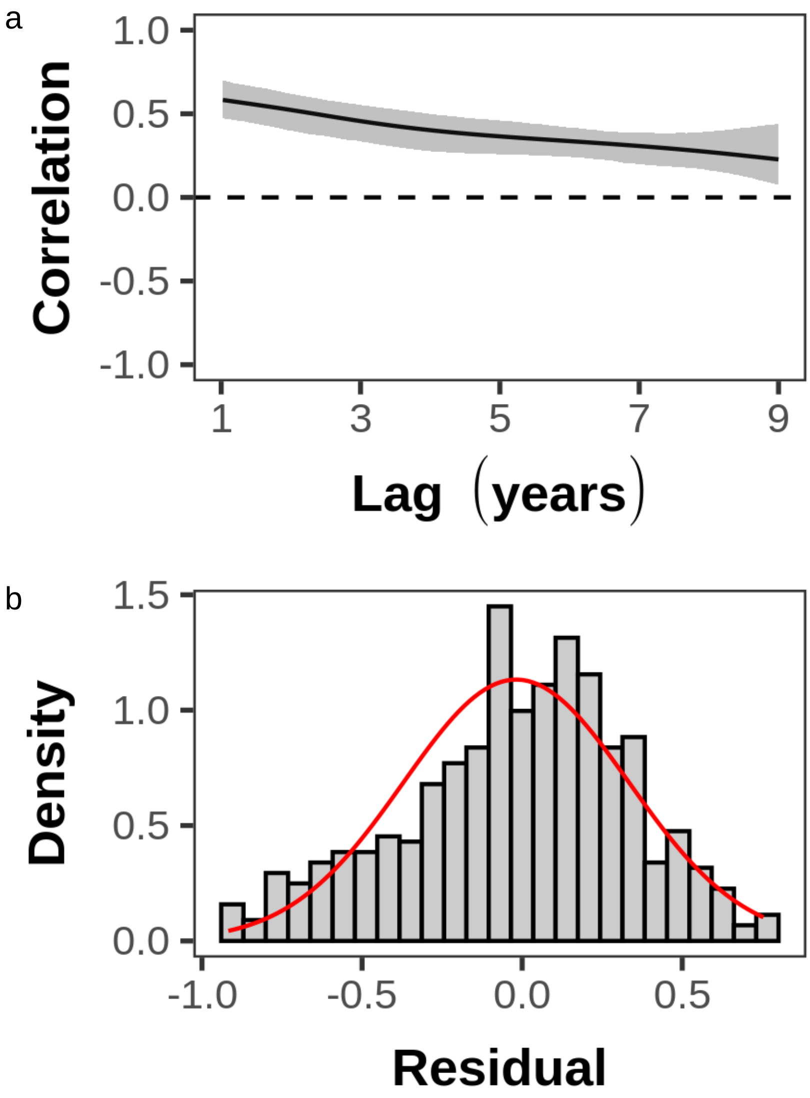

Supplementary Table S1. Estimated elephant populations on MIKE sites by year. Sources refer to reference numbers.

Country	Site	MIKE code	Year	Estimate	Source
Benin	Pendjari	PDJ	2003	713	1
Benin	Pendjari	PDJ	2015	1202	2
Benin	W du Burkina	WBF	2003	740	1
Benin	W du Burkina	WBF	2015	1040	2
Benin	W du Bénin	WBJ	2003	54	1
Benin	W du Bénin	WBJ	2015	521	2
Benin	W du Niger	WNE	2003	85	1
Benin	W du Niger	WNE	2015	0	2
Botswana	Chobe	CHO	2002	35317	2
Botswana	Chobe	CHO	2004	35570	2
Botswana	Chobe	CHO	2006	43417	2
Botswana	Chobe	CHO	2010	26609	3
Botswana	Chobe	CHO	2011	21061	2
Botswana	Chobe	CHO	2012	29468	2
Botswana	Chobe	CHO	2013	26598	2
Botswana	Chobe	CHO	2014	17448	4
Botswana	Chobe	CHO	2018	15404	5
Burkina Faso	Nazinga	NAZ	2003	548	1
Burkina Faso	Nazinga	NAZ	2012	893	6
Cameroon	Boumba-Bek	BBK	2004	318	6
Cameroon	Boumba-Bek	BBK	2008	2062	6
Cameroon	Boumba-Bek	BBK	2012	2062	6
Cameroon	Boumba-Bek	BBK	2015	143	6
Cameroon	Waza	WAZ	2002	987.5	7
Cameroon	Waza	WAZ	2007	246	6
Cent. Afr. Republic	Bangassou	BGS	1998	1600	6
Cent. Afr. Republic	Bangassou	BGS	2004	750	6
Cent. Afr. Republic	Dzanga-Sangha	DZA	2005	1035	6
Cent. Afr. Republic	Dzanga-Sangha	DZA	2012	797	6
Cent. Afr. Republic	Sangba	SGB	2005	708	8
Cent. Afr. Republic	Sangba	SGB	2010	68	9
Chad	Zakouma	ZAK	2002	4351	2
Chad	Zakouma	ZAK	2005	3885	2
Chad	Zakouma	ZAK	2006	3020	2
Chad	Zakouma	ZAK	2008	937	2
Chad	Zakouma	ZAK	2009	617	2
Chad	Zakouma	ZAK	2010	542	2
Chad	Zakouma	ZAK	2011	454	2
Chad	Zakouma	ZAK	2012	444	2

Country	Site	MIKE code	Year	Estimate	Source
Chad	Zakouma	ZAK	2014	443	[2]
Congo	Nouabale-Ndoki	NDK	2003	2652	[8]
Congo	Nouabale-Ndoki	NDK	2011	2324	[6]
Congo	Odzala-Kokoua	ODZ	2005	7460	[7]
Congo	Odzala-Kokoua	ODZ	2012	9292	[6]
Cote d'Ivoire	Marahoué	MAR	2002	159	[6]
Cote d'Ivoire	Marahoué	MAR	2010	0	[6]
Cote d'Ivoire	Taï	TAI	2002	53	[7]
Cote d'Ivoire	Taï	TAI	2010	189	[7]
DR Congo	Garamba	GAR	2007	3636	[2]
DR Congo	Garamba	GAR	2012	1717	[2]
DR Congo	Garamba	GAR	2014	1156	[2]
DR Congo	Okapi	OKP	2006	2698	[7]
DR Congo	Okapi	OKP	2011	1701	[6]
DR Congo	Salonga	SAL	2004	1186	[7]
DR Congo	Virunga	VIR	2003	286	[2]
DR Congo	Virunga	VIR	2006	348	[2]
DR Congo	Virunga	VIR	2010	347	[2]
DR Congo	Virunga	VIR	2014	35	[2]
Eritrea	Gash-Setit	GSH	2002	83	[6]
Eritrea	Gash-Setit	GSH	2006	104	[6]
Eritrea	Gash-Setit	GSH	2012	120	[6]
Eritrea	Gash-Setit	GSH	2014	100	[6]
Ethiopia	Babille	BBL	1998	65	[10]
Ethiopia	Babille	BBL	2005	264	[6]
Ethiopia	Babille	BBL	2009	324	[6]
Ethiopia	Babille	BBL	2014	250	[6]
Gabon	Lopé	LOP	2005	2350	[6]
Gabon	Lopé	LOP	2009	4142	[6]
Gabon	Minkébé	MKB	2004	34128	[11]
Gabon	Minkébé	MKB	2015	6956	[11]
Ghana	Kakum	KAK	2000	294	[7]
Ghana	Kakum	KAK	2004	164	[6]
Ghana	Mole	MOL	2004	259	[12]
Ghana	Mole	MOL	2006	401	[6]
Guinea	Ziama	ZIA	2004	214	[7]
Guinea	Ziama	ZIA	2011	101	[7]
Kenya	Mount Elgon	EGK	2002	139	[6]
Kenya	Mount Elgon	EGK	2015	200	[6]
Kenya	Samburu Laikipia	SBR	2002	5458	[13]
Kenya	Samburu Laikipia	SBR	2012	6365	[14]
Kenya	Samburu Laikipia	SBR	2014	7134	[2]
Kenya	Tsavo	TSV	2005	10356	[15]

Country	Site	MIKE code	Year	Estimate	Source
Kenya	Tsavo	TSV	2008	11592	16
Kenya	Tsavo	TSV	2011	12000	17
Kenya	Tsavo	TSV	2014	11144	7
Kenya	Tsavo	TSV	2014	13794	2
Liberia	Sapo	SAP	2009	124	7
Malawi	Kasungu	KSG	2005	58	6
Malawi	Kasungu	KSG	2014	40	2
Mali	Gourma	GOU	2002	322	18
Mali	Gourma	GOU	2007	344	19
Mali	Gourma	GOU	2015	253	2
Mozambique	Limpopo	LPP	2010	1425	20
Mozambique	Limpopo	LPP	2014	1081	2
Mozambique	Magoe	MAG	2003	1628	21
Mozambique	Magoe	MAG	2010	1465	22
Mozambique	Magoe	MAG	2014	1051	2
Mozambique	Niassa	NIA	2002	13061	2
Mozambique	Niassa	NIA	2004	12477	2
Mozambique	Niassa	NIA	2006	11833	2
Mozambique	Niassa	NIA	2009	20364	2
Mozambique	Niassa	NIA	2011	12029	2
Mozambique	Niassa	NIA	2014	4441	2
Namibia	Etosha	ETO	2002	2417	6
Namibia	Etosha	ETO	2004	2057	6
Namibia	Etosha	ETO	2011	3378	6
Namibia	Etosha	ETO	2012	2810	7
Namibia	Etosha	ETO	2015	2911	6
Namibia	Zambezi	ZBZ	2013	4435	23
Namibia	Zambezi	ZBZ	2015	8057	6
Nigeria	Yankari	YKR	1999	328	12
Nigeria	Yankari	YKR	2006	348	7
Nigeria	Yankari	YKR	2011	221.5	7
Rwanda	Akagera	AKG	2002	34	6
Rwanda	Akagera	AKG	2013	88	6
Senegal	Niokolo-Koba	NKK	2002	26	12
Senegal	Niokolo-Koba	NKK	2006	6	12
South Africa	Kruger	KRU	2003	11672	2
South Africa	Kruger	KRU	2004	11483	2
South Africa	Kruger	KRU	2005	12467	2
South Africa	Kruger	KRU	2006	12427	2
South Africa	Kruger	KRU	2007	13050	2
South Africa	Kruger	KRU	2008	12930	2
South Africa	Kruger	KRU	2009	13573	2
South Africa	Kruger	KRU	2010	13750	2

Country	Site	MIKE code	Year	Estimate	Source
South Africa	Kruger	KRU	2011	14454	2
South Africa	Kruger	KRU	2012	16571	2
South Africa	Kruger	KRU	2015	17086	2
Tanzania	Katavi Rukwa	KTV	2002	5160	6
Tanzania	Katavi Rukwa	KTV	2007	5302	6
Tanzania	Katavi Rukwa	KTV	2009	5359	6
Tanzania	Katavi Rukwa	KTV	2014	3250	2
Tanzania	Mkomazi	MKZ	2002	63	17
Tanzania	Mkomazi	MKZ	2005	41	17
Tanzania	Mkomazi	MKZ	2008	8	17
Tanzania	Mkomazi	MKZ	2011	256	7
Tanzania	Mkomazi	MKZ	2014	59	6
Tanzania	Ruaha Rungwa	RHR	2013	18562	24
Tanzania	Ruaha Rungwa	RHR	2014	7769	2
Tanzania	Selous Mikumi	SEL	2002	41021	6
Tanzania	Selous Mikumi	SEL	2013	10546	25
Tanzania	Selous Mikumi	SEL	2014	12304	2
Tanzania	Tarangire-Manyara	TGR	2000	2370	2
Tanzania	Tarangire-Manyara	TGR	2004	1886	2
Tanzania	Tarangire-Manyara	TGR	2006	1755	2
Tanzania	Tarangire-Manyara	TGR	2009	2245	2
Tanzania	Tarangire-Manyara	TGR	2014	4095	2
Togo	Fazao Mafakassa	FAZ	2002	61	6
Togo	Fazao Mafakassa	FAZ	2016	80	6
Uganda	Murchison Falls	MCH	2005	516	2
Uganda	Murchison Falls	MCH	2010	904	2
Uganda	Murchison Falls	MCH	2012	1617	2
Uganda	Murchison Falls	MCH	2014	1330	2
Uganda	Queen Elizabeth	QEZ	2000	1086	2
Uganda	Queen Elizabeth	QEZ	2002	998	2
Uganda	Queen Elizabeth	QEZ	2004	2497	2
Uganda	Queen Elizabeth	QEZ	2006	2959	2
Uganda	Queen Elizabeth	QEZ	2010	2502	2
Uganda	Queen Elizabeth	QEZ	2012	3018	2
Uganda	Queen Elizabeth	QEZ	2014	2913	2
Zambia	South Luangwa	SLW	2002	4459	6
Zambia	South Luangwa	SLW	2009	4419	26
Zambia	South Luangwa	SLW	2011	3855	6
Zambia	South Luangwa	SLW	2012	2813	6
Zambia	South Luangwa	SLW	2015	3302	2
Zimbabwe	Chewore	CHE	2001	5741	27
Zimbabwe	Chewore	CHE	2010	5048	28
Zimbabwe	Chewore	CHE	2014	3303	2

Country	Site	MIKE code	Year	Estimate	Source
Zimbabwe	Nyami Nyami	NYA	2001	2372	[6]
Zimbabwe	Nyami Nyami	NYA	2006	3715	[6]
Zimbabwe	Nyami Nyami	NYA	2014	411	[2]

References

1. Bouché, P., Lungren, C., Hien, B. & Omondi, P. *Aerial total count of the "W"-Arly-Pendjari-Oti-Mandouri-Keran (WAPOK) ecosystem in west Africa*. CITES–MIKE/ECOPAS/PAUCOF (2004).
2. Chase, M. J. *et al.* Continent-wide survey reveals massive decline in african savannah elephants. *PeerJ* **4**,e2354; 10.7717/peerj.2354 (2016).
3. Chase, M. *Dry season fixed-wing aerial survey of elephants and wildlife in northern Botswana, September - November 2010*. Elephants Without Borders, http://elephantswithoutborders.org/downloadspapers/EWB 2010 BW Survey.pdf (2011).
4. Chase, M. *et al. Dry season aerial survey of elephants and wildlife in northern Botswana, July - October 2014*. Elephants Without Borders (2015).
5. Chase, M. J. & Schlossberg, S. *Dry season aerial survey of elephants and wildlife in northern Botswana*. Elephants Without Borders (2019).
6. IUCN. Elephant database. http://www.elephantdatabase.org/preview_report/2013_africa_final/Loxodonta_africana/2013/Africa (2013).
7. Thouless, C. R. *et al. African elephant status report 2016: an update from the African Elephant Database*. Occasional paper of the IUCN Species Survival Commission no. 60 (2016).

8. Blake, S. *Central African forests: final report on population surveys (2003-2004). March 2005*. CITES MIKE (2005).
9. Bouché, P. *Inventaire aerien 2010 des grands mammifères dans le nord de la République Centafricaine*. http://africanelephantdatabase.org/system/population_submission_attachments/files/000/000/116/original/svyFCCFSGB2010AS.pdf (2010).
10. Blanc, J. J. *et al. African elephant status report 2002: an update from the African Elephant Database*. IUCN/SSC African Elephant Specialist Group (2003).
11. Poulsen, J. R. *et al.* Poaching empties critical central African wilderness of forest elephants. *Current Biology* **27**,R134–R135; 10.1016/j.cub.2017.01.023 (2017).
12. Blanc, J. J. *et al. African elephant status report 2007: an update from the African Elephant Database*. IUCN/SSC African Elephant Specialist Group (2007).
13. Omondi, P., Bitok, E., Kahindi, O. & Mayienda, R. *Total Aerial Count of Elephants in Samburu-Laikipia EcoSystem: June 2002*. Kenya Wildlife Service, Elephant Programme (2002).
14. Ngene, S. *et al. Total aerial count of elephants, Grevy's zebra and other large mammals in Laikipia-Samburu-Marsabit ecosystem in (November 2012)*. Kenya Wildlife Service (2013).
15. Omondi, P. & Bitok, E. *Total aerial count of elephants, buffalo and other species in the Tsavo/Mkomazi ecosystem*. Kenya Wildlife Service, Elephant Programme (2005).
16. Omondi, P., Bitok, E., Mukeka, J., Mayienda, R. & Litoroh, M. *Total aerial count of elephants and other large mammal species of Tsavo/Mkomazi ecosystem*. Kenya Wildlife Service (2008).

17. Ngene, S. *et al. Total aerial census of elephants and other large mammals in the Tsavo-Mkomazi ecosystem*. Kenya Wildlife Service (2011).
18. Blake, S., Bouché, P., Rasmussen, H., Orlando, A. & Douglas-Hamilton, I. *The last Sahelian elephants: ranging behavior, population status, and recent history of the desert elephants of Mali*. Save the Elephants (2003).
19. Bouché, P. *Dénombrement des éléphants du Gourma*. IUCN & Projet de Conservation et de Valorisation de la Biodiversité du Gourma et des éléphants Republique du Mali, http://africanelephantdatabase.org/population_submission_attachments/148 (2007).
20. Stephenson, A. *Parque Nacionale do Limpopo aerial wildlife census*. Wildlife Management Services (2010).
21. Dunham, K. M. *Aerial survey of elephants and other large herbivores in the Zambezi heartland (Zimbabwe, Mozambique, Zambia) 2003*. Africa Wildlife Foundation (2004).
22. Dunham, K. M. *Aerial survey of elephants and other large herbivores south of Lake Cabora Bassa, Mozambique: 2010*. AGRECO G.E.I.E., http://africanelephantdatabase.org/system/population_submission_attachments/files/000/000/069/original/svyFSMZCBA2010AS.pdf (2010).
23. Craig, G. C. & Gibson, D. St. C. *Aerial survey of elephants and other wildlife in the Caprivi, May-June 2013*. World Wildlife Fund, http://africanelephantdatabase.org/system/population_submission_attachments/files/000/000/367/original/svyFSNACAP2013AS.pdf (2013).
24. TAWIRI. *Aerial census of large animals in the Ruaha-Rungwa Ecosystem, dry season 2013*. (2013).

25. TAWIRI. *Aerial census of large animals in the Selous-Mikumi Ecosystem, Dry Season 2013*. (2013).

26. WCS Flight Programme. *Aerial survey report: Luangwa Valley 2009*. Wildlife Conservation Society (2009).

27. Mackie, C. S. *Aerial census of elephants and other large herbivores in the Zambezi Valley, Zimbabwe: 2001*. DNPWM/WWF (2002).

28. Kuvawoga, P. T. & Gandiwa, E. *Aerial survey of elephants and other large herbivores in Chewore Safari Area, Zambezi Valley: 2010*. CITES MIKE Programme, http://africanelephantdatabase.org/population_submission_attachments/455 (2011).

R Code to Conduct Analyses in the Paper

```
# Following is code to run the analyses in "State-space models reveal a continuing elephant poaching
# problem in most of Africa" by Schlossberg et al.

require(MASS)     # needed to use ginv(), the generalized inverse of a matrix
require(KFAS)     # package for running Kalman filter/smoother
require(car)      # needed to access a fast wcrossprod function
require(dplyr)
require(tidyr)
require(tibble)
require(psych)
require(ggplot2)
require(purrr)
require(furrr)
require(zoo)
options(stringsAsFactors = F)

###############################################################################################
#                                    Import and format data                                  #
###############################################################################################

#load MIKE data, available from https://www.cites.org/eng/prog/mike/index.php#Access%20to%20MIKE%20Data
# column names: "continent","region","regionID","country","ccode","site","site.name","year","tot","ill"
# tot = total number of carcasses reported; ill = number of illegally killed carcasses
mike=read.csv("mike_data.csv")

#load elephant population estimates (available in Supplementary Table S1) and join with MIKE data
ests=read.csv("population_estimates.csv") %>% as_tibble
mike.ests=full_join(mike, ests %>% select(-country,-site.name),by=c("site","year"))

#remove 2 sites with no elephant population size estimates
mike.ests= mike.ests %>% group_by(site) %>% filter(!all(is.na(estimate))) %>% ungroup
```

```
#linear interpolation of elephant population estimates for missing years

mike.ests=mike.ests %>% arrange(country,site,year) %>% group_by(site) %>% nest()
interpolate=function(df) {
   df.loc=df
   #add any missing years for 2003-2018
   df.loc=complete(df.loc,year=2003:2018,fill=
      list(region=df.loc$region[1],regionID=df.loc$regionID[1],site.name=df.loc$site.name[1],
      country=df.loc$country[1])) %>% arrange(desc(year))
   #if only one esetimate is available, use it for all years
   if(sum(!is.na(df.loc$estimate))==1) {
      df.loc$estimate.interp=df.loc$estimate[!is.na(df.loc$estimate)]
      return(df.loc)
   }
   nas=which(is.na(df.loc$estimate))
   ests.loc=df.loc$estimate
   interp=na.approx(zoo(x=df.loc$estimate),xout=nas) #Linear interpolation
   ests.loc[index(interp)]=interp
   #assign values prior to the earliest survey or after the latest survey
   ests.loc[(1:length(ests.loc)) > max(which(!is.na(df.loc$estimate)))]=
      ests.loc[max(which(!is.na(df.loc$estimate)))]
   ests.loc[(1:length(ests.loc)) < min(which(!is.na(df.loc$estimate)))]=
      ests.loc[min(which(!is.na(df.loc$estimate)))]
   df.loc$estimate.interp=ests.loc
   df.loc=filter(df.loc,year>2002) #2003 is the earliest year for analysis
   return(df.loc)
}
#run the interpolation and clean up
mike.ests=mike.ests %>% mutate(data=map(data, interpolate)) %>% unnest(data)
rm(ests,mike,interpolate)

#replace missing value for total carcasses with 10 (this is a placeholder and does not affect results;
# model cannot accept missing values for denominator)
mike.ests=mike.ests %>% mutate(tot=replace(tot,tot==0,NA))
```

```
#########################################################################################################
#                                         Run the Kalman smoother                                       #
#########################################################################################################

#function to run Kalman smoother for one region

runmods=function(rgn, min.pre=4, min.post=4) {
   #rgn = one regionID from "mike.ests" as a string
   #min.pre = minimum number of years with data for 2003-2011 to include a site in the analysis
   #min.post = minimum number of years with data for 2011-2018 to include a site in the analysis

   #select data from "mike.ests"
   mike=mike.ests %>%   filter(regionID==rgn) %>%
      group_by(site) %>%
      filter(sum(!is.na(tot[year<=2011]))>=min.pre & sum(!is.na(tot[year>=2011]))>=min.post) %>%
      ungroup() %>%
      mutate(tot=replace_na(tot,10))

   #create matrices with numbers of illegally killed and all carcasses; rows = years, columns = sites
   df.mv=mike %>% pivot_wider(values_from=c(ill,tot),names_from=site,id_cols=year)
   ill=df.mv %>% select_at(vars(matches("ill"))) %>% as.matrix #illegally killed carcasses
   tot=df.mv %>% select_at(vars(matches("tot"))) %>% as.matrix #all carcasses
   n_sites=length(unique(mike$site))

   #set up model
   model = SSModel(ill ~ -1+
      SSMcustom(Z = diag(1, n_sites), T = diag(1, n_sites),
            Q = diag(NA,n_sites),
            P1 = matrix(NA, n_sites,n_sites)),distribution = "binomial",u = tot)
   updatefn <- function(pars, model, ...) {
      Q <- diag(pars[1:n_sites])
      model["Q", etas = "custom"] <- model["P1", states = "custom"] <-crossprod(Q)
      model
   }

   #create initial estimates for sPIKE using actual PIKE values
   pike.init=ill/tot
   #because estimation is on the logit scale, 1's and 0's are not allowed in initial values
   pike.init[pike.init==1]=.9
   pike.init[is.na(pike.init)|pike.init==0]=.1
   init <- chol(cov(logit(pike.init)))
```

```
   #calculate initial values for process error variance
   fitinit <- fitSSM(model, updatefn = updatefn,
      inits = rep(c(log(diag(init)), init[upper.tri(init)]), 2),
      method = "BFGS")

   #calculate final values for covariance
   fit <- fitSSM(model, updatefn = updatefn, inits = fitinit$optim.out$par,
      method = "BFGS", nsim=250)

   #run filtering and smoothing algorithm (by default, KFS function filters and smooths)
   out=KFS(fit$model, nsim=1000)

   return(list(model=out, data=mike))
}

#run models for each region using parallel processing to reduce time required
plan(multisession,workers=4) #set up cluster
models=future_map(c("FS","FE","FW","FC"),runmods,.progress=T)
names(models)=c("FS","FE","FW","FC")


###############################################################################################
#                                    Summarize model results                                  #
###############################################################################################

#get predicted sPIKE values by site and year
   #fit = estimate on logit scale
   #lwr/upr = lower and upper 95% confidence limits
   #se.fit = standard error of estimate on logit scale

sPIKE_by_site = map_dfr(models,~{
   pred=fitted(.x$model) %>% as.matrix %>% logit
   n=dim(.x$model$V)[1]
   ses=map(1:n, function(site) sqrt(.x$model$V[site,site,])) %>% do.call(cbind,.)
   tibble(fit=gdata::unmatrix(pred),se.fit=gdata::unmatrix(ses)) %>% cbind(.x$data,.) %>%
      mutate(lwr=fit-1.96*se.fit,upr=fit+1.96*se.fit)
})
```

```
#plot predicted and actual values for individual sites by region on probability (not logit) scale

plot.region=function(reg) { #reg = 2-letter regionID value
   df=sPIKE_by_site %>% mutate_at(vars(matches("^fit$|lwr|upr")),psych::logistic) %>%
      filter(regionID==reg)
   ggplot(df,aes(year,fit))+
      geom_line(size=.25)+geom_ribbon(aes(ymin=lwr,ymax=upr),alpha=.3)+
      geom_point(aes(y=ill/tot,size=tot),fill=NA,shape=1,stroke=.2)+
            scale_size_area("Carcasses",max_size=3,breaks=c(1,10,100,300))+
      facet_wrap(~site.name,ncol=3)+theme_bw(7)+
      theme(aspect.ratio=0.7,panel.grid=element_blank(),legend.key.height=unit(0.7,"lines"))+
      xlab(expression(bold(Year)))+ylab(expression(bold(PIKE)))
}

plot.region("FS")+theme(legend.position="bottom")
plot.region("FE")+theme(legend.position=c(.8,.1))
plot.region("FC")+theme(legend.position=c(.83,.12))
plot.region("FW")+theme(legend.position=c(.72,.15))


###############################################################################################
#                                  Calculate regional sPIKE                                   #
###############################################################################################

#regional predicted values from KFAS model
sPIKE_by_region=

   #duplicate site data.frame to create a new region for entire continent with regionID = "AFR"
   bind_rows(sPIKE_by_site, sPIKE_by_site %>% mutate(region="All Africa",regionID="AFR")) %>%
   group_by(region, regionID, year) %>%

   #calculate weight for combining sites = (est. # of live elepants)/(sum of est. # of live elephants)
   mutate(weight=estimate.interp/sum(estimate.interp)) %>%
   summarize(n=sum(tot,na.rm=T),sPIKE=sum(logistic(fit)*weight),
      se_logit=sqrt(sum(se.fit^2*weight^2)), #logit scale std. error of sPIKE
      se_p=sPIKE*(1-sPIKE)*se_logit, #probability scale std. error of sPIKE via delta method
      #calculate sPIKE +/- 1 SE on probability scale
      sPIKE_se_upper=logistic(logit(sPIKE)+se_logit),sPIKE_se_lower=logistic(logit(sPIKE)-se_logit),
      #calculate sPIKE 95% confidence limits on probability scale
      lcl_p=logistic(logit(sPIKE)-se_logit*1.96),ucl_p=logistic(logit(sPIKE)+se_logit*1.96)) %>%
   ungroup()
```

```
#plot regional results including confidence regions for +/- 1 SE and +/- 95% CI

gplot(sPIKE_by_region, aes(year, sPIKE)) + geom_line()+
   geom_ribbon(aes(ymin=lcl_p,ymax=ucl_p),alpha=.3)+
   geom_ribbon(aes(ymin=sPIKE_se_lower,ymax=sPIKE_se_upper),alpha=.5)+
   facet_wrap(~region)+theme_bw(13)+theme(aspect.ratio=.7,panel.grid=element_blank())+
   xlab(expression(bold(Year)))+ylab(expression(bold(sPIKE)))


###############################################################################################
#                              Calculate trends in sPIKE by region                            #
###############################################################################################

#Feasible generalized least squares method from Lewis and Linzer (2005)
tr=function(x) { sum(diag(x)) } #trace of a square matrix

#function to run FGLS model

edvreg <- function(mod,omegasq,proportional=FALSE) {
   #  edvreg
   #     mod: Regression formula
   #
   #     omegasq: Nx1 vector of estimated sampling/error variances of the dependent variable
   #
   #     proportional: "True" if omegasq is only proportional to the sampling/error variances of
   #                   of the dependent variables
   #
   # Example call: res <- edvreg( y ~ x, omegasq = est.var.y )
   #
   # Some housekeeping stolen from John Fox's corchrane-orcutt implementation
   X <- model.matrix(mod)
   xnames <- colnames(X)
   y <- model.response(model.frame(mod))

   # When omegasq is known up to a proportion (for example 1/N_i) ...
   if (proportional) {
        residsq <- (resid(lm(y~X-1)))^2
      steptwo <- lm(residsq~omegasq)
        zeroeps <- FALSE
      if (coef(steptwo)[1] < 0) {
            warning("Variance of epsilon estimated to be negative! Setting to 0...")
```

```
            steptwo <- lm(residsq~0+omegasq)
            zeroeps <- TRUE
      }
      predval <- fitted.values(steptwo)
      if (sum(predval<0) < 0) {
         warning("Negative omegaSq estimates encountered, setting to 0.0001")
               predval[predval<0] <- 0.0001 # error check against very rare possibility of negative weight
      }
      model.FGLS <- lm(y~X-1,weights=(1/predval))
       model.FGLS$vareps <- ifelse(zeroeps,0,steptwo$coefficients[1])
       model.FGLS$omegasqFactor <- ifelse(zeroeps,steptwo$coefficients[1],steptwo$coefficients[2])
      model.FGLS$predval <- predval
   }
    # When omegasq is known exactly...
    else {
        N <- nrow(X)
        sigmahatsq <- (sum((residuals(lm(y~X-1)))^2) - sum(omegasq) +
                  tr(ginv(crossprod(X)) %*% wcrossprod(X,X,w=omegasq)))/(N-ncol(X)-1)
        if (sigmahatsq < 0) {
           warning(sprintf("SigmaHatSq estimated to be %7.4f, setting to 0...",sigmahatsq))
               sigmahatsq <- 0.0
        }
        w <- 1/(omegasq+sigmahatsq)
        model.FGLS <- lm(y~X-1, weights=w)
        model.FGLS$sigmahatsq <- sigmahatsq
    }
    names(model.FGLS$coefficients) <- xnames
    model.FGLS
}

#run models and summarize results for state-space models

trends_region=sPIKE_by_region %>% mutate(time=ifelse(year<=2010,"2003-2010","2011-2018")) %>%
   group_by(region,time) %>% nest %>%
   mutate(reg=map(data,~broom::tidy(edvreg((.x$sPIKE)~.x$year,.x$se_p^2)) %>% slice(2))) %>%
   unnest(cols=reg) %>% ungroup() %>% select(-data,-term) %>% mutate(type="State-space")
```
